# Disentangling Genuine Semantic Stroop Effects in Reading from Contingency Effects: On the Need for Two Neutral Baselines

**DOI:** 10.3389/fpsyg.2016.00386

**Published:** 2016-03-17

**Authors:** Eric Lorentz, Tessa McKibben, Chelsea Ekstrand, Layla Gould, Kathryn Anton, Ron Borowsky

**Affiliations:** Psychology, University of SaskatchewanSaskatoon, SK, Canada

**Keywords:** semantics, stroop effect, contingency learning, color associates, pseudohomophones, reading

## Abstract

The automaticity of reading is often explored through the Stroop effect, whereby color-naming is affected by color words. Color associates (e.g., “sky”) also produce a Stroop effect, suggesting that automatic reading occurs through to the level of semantics, even when reading sub-lexically (e.g., the pseudohomophone “skigh”). However, several previous experiments have confounded congruency with contingency learning, whereby faster responding occurs for more frequent stimuli. Contingency effects reflect a higher frequency-pairing of the word with a font color in the congruent condition than in the incongruent condition due to the limited set of congruent pairings. To determine the extent to which the Stroop effect can be attributed to contingency learning of font colors paired with lexical (word-level) and sub-lexical (phonetically decoded) letter strings, as well as assess facilitation and interference relative to contingency effects, we developed two neutral baselines: each one matched on pair-frequency for congruent and incongruent color words. In Experiments 1 and 3, color words (e.g., “blue”) and their pseudohomophones (e.g., “bloo”) produced significant facilitation and interference relative to neutral baselines, regardless of whether the onset (i.e., first phoneme) was matched to the color words. Color associates (e.g., “ocean”) and their pseudohomophones (e.g., “oshin”), however, showed no significant facilitation or interference relative to onset matched neutral baselines (Experiment 2). When onsets were unmatched, color associate words produced consistent facilitation on RT (e.g., “ocean” vs. “dozen”), but pseudohomophones (e.g., “oshin” vs. “duhzen”) failed to produce facilitation or interference. Our findings suggest that the Stroop effects for color and associated stimuli are sensitive to the type of neutral baseline used, as well as stimulus type (word vs. pseudohomophone). In general, contingency learning plays a large role when repeating congruent items more than incongruent items, but appropriate pair-frequency matched neutral baselines allow for the assessment of genuine facilitation and interference. Using such baselines, we found reading processes proceed to a semantic level for familiar words, but not pseudohomophones (i.e., phonetic decoding). Such assessment is critical for separating the effects of genuine congruency from contingency during automatic word reading in the Stroop task, and when used with color associates, isolates the semantic contribution.

## Introduction

Since its instantiation over 80 years ago, the Stroop effect (Stroop, 1935) has been widely investigated as a basic and robust phenomenon in cognitive psychology, and yet the source of this effect is still in contention (Melara and Algom, 2003; Levin and Tzelgov, 2014; see MacLeod, 1991 for a comprehensive review). The Stroop effect refers to the phenomenon wherein naming the font color of a congruent color word (CW; e.g., “blue” in the font color blue) shows speeded responding relative to an incongruent CW (e.g., “red” in the font color blue), despite the instructions to not read the words (i.e., unintentional, automatic word reading). One can also investigate, relative to a neutral baseline, facilitation in the congruent condition, and/or interference in the incongruent condition. Typically, greater interference is found than facilitation (Cheesman and Merikle, 1984; MacLeod, 1991; Lindsay and Jacoby, 1994).

Among the hypotheses proposed to account for Stroop effects, many have argued that both semantic conflict and response conflict make up the basis of Stroop effects (MacLeod, 1991; Botvinick et al., 2001; De Houwer, 2003; Kane and Engle, 2003; Roelofs, 2010; Anton et al., 2014). In the semantic processing account, multiple meanings from word and font color sources compete for processing resources in incongruent trials and converge to facilitate processing in congruent trials at a semantic input locus. In the response conflict account, the word stimulus evokes a learned response that is immediately brought to the output stage, which facilitates responding when congruent with the correct response, and interferes with responding when incongruent with the correct response. As semantic and response conflict models are the prevailing theoretical accounts of the Stroop effect, a significant literature has been developed around stimuli that evoke semantic conflict, but not response conflict, in order to tease apart the influences of the two. Demonstrating evidence for the semantic account, Klein (1964) first used color associates (CAs), words that are strongly associated with a particular color, (e.g., “sky” for the color blue) as stimuli in the Stroop task and found greater interference with incongruent CAs relative to common words unrelated to color (e.g., put). Soon after, Dalrymple-Alford (1972) extended these effects by including a congruent condition, presenting each word twice in each color, which revealed facilitation with congruent CAs in addition to interference with incongruent CAs, but that facilitation and interference with CAs was not as large as with CWs. Arguably, this was because CAs do not elicit response competition (see also Risko et al., 2006, on response set effects with CAs). As CAs appears to demonstrate significant facilitation and interference, there is strong support for semantic processing playing a significant role in the Stroop effect.

The automaticity with which word meaning is accessed during color-naming has also been investigated using CW pseudohomophones (CWPHs; e.g., green spelled “ghrean”), which require phonetic decoding instead of whole word retrieval for semantic access (Anton et al., 2014). For instance, Dennis and Newstead (1981) had participants name the color of CWs, CWPHs and common word PHs matched to CWs for first letter and onset (i.e., first phoneme, e.g., grief spelled “greef”) when the font color was incongruent with the word (i.e., “greef” was never presented in green) and found significant interference for both CWs and CWPHs relative to neutral PHs. A second experiment included congruent stimuli (i.e., “green,” “grean,” and “greef” presented in green font) shown three times in their congruent color and three times in different incongruent colors. The results showed faster responding for congruent CWs, CWPHs, and neutral word PHs relative to incongruent stimuli, supporting Stroop effects even when forcing the use of phonetic decoding. Moreover, while incongruent CWs showed more interference than CWPHs, congruent CWs did not show more facilitation than congruent CWPHs, which the authors attribute to fast recognition of onset matching, and the slow process of phonetic decoding. That is, onset matching slowed down incongruent stimuli enough that phonetic decoding would further interfere in the incongruent condition, but fast recognition of onset in the congruent condition would show identical results between CWs and CWPHs.

A noteworthy aspect of the methodology used by Dennis and Newstead (1981) is that each congruent letter string was presented three times, whereas incongruent letter strings were only presented once in three different colors. When the congruent stimuli, including the neutral PHs, were responded to faster than incongruent stimuli, the authors attributed this finding to the rapid recognition of each word's onset (which matched the first letter of the color response in the congruent condition). An equally plausible explanation, however, is that participants learned the correlation created between stimulus and response (also described as covariation; see Melara and Algom, 2003), given that congruent trials are repeated more often than incongruent trials (i.e., contingency learning). That is, “grean” and “greef” elicited the response “green” 50% of the time (high pair-frequency) and all other possible responses ~17% of the time (low pair-frequency). This alternative explanation may, in fact, be more plausible as color-naming shows faster responding with high pair-frequency stimuli than low pair-frequency stimuli, even when using neutral words without onset matching (e.g., Schmidt et al., 2007).

In the context of the classic Stroop task, certain stimulus-response frequencies occur when equating the proportion of congruent and incongruent stimuli. Consider balancing the number of congruent and incongruent items in an experiment with nine colors: for each congruent item (e.g., “blue” in the font color blue), there are eight incongruent items (i.e., “blue” in the eight remaining colors). Thus, the word blue would be paired with the font color blue eight times more often than any other color in order to equate the number of congruent and incongruent trials, and this could lead to significant contingency learning, which may wrongly be interpreted as genuine Stroop effects. In light of this methodological concern, Schmidt and Besner (2008) studied both contingency and congruency effects together by pairing each font color with exactly one congruent and one incongruent color word. Each color was presented with a proportion of either 75, 50, or 25% congruent trials. With this design, color word, and font color associations could be learned such that the word would predict the font color response (contingency) with either 75 or 50% accuracy for both congruent and incongruent items. Their results indicated slowed responses for incongruent trials relative to congruent trials (congruency effect), but more interestingly that the predictive nature of color words produced faster RTs for 75% predictive color words than 50% predictive color words (contingency learning). These results suggest that the Stroop effect may be attributable to both congruency effects and contingency effects, and that contingency learning plays a significant role in the Stroop task.

Contingency effects reveal the tendency to detect covariation between both relevant and irrelevant dimensions of the Stroop task (Melara and Algom, 2003). When including both congruent and incongruent trials, prior research has shown a proclivity to equate the number of congruent and incongruent trials in an orthogonal design (see MacLeod, 1991, for review), which tends to inflate the frequency of congruent color/word pairings relative to the frequency of incongruent color/word less pairings. Detecting this covariation encourages attention to word identity, and may benefit higher frequency congruent trials and hinder frequent incongruent trials, enhancing the magnitude of the measured Stroop effect that is attributed to congruency.

A number of research programmes have omitted congruent pairings altogether to avoid this artifact, comparing only equally frequent incongruent and neutral color/word pairings (e.g., Manwell et al., 2004; Risko et al., 2006; Augustinova et al., 2014). These studies provide a measure of the interference produced by incongruent color words and CAs. However, it is worth noting that they also create a negative covariation, whereby word identity still predicts responding (i.e., the word blue may be predictive of a “red” response), and can still encourage attention to the word. This design also foregoes the study of facilitation in the Stroop task, and how the inclusion of congruent stimuli can influence the context of the Stroop task as a whole (e.g., Dennis and Newstead, 1981). One solution includes presenting all congruent and incongruent color/word pairings equally frequently, but at the cost of equating the proportion of congruent and incongruent trials (e.g., Dishon-Berkovits and Algom, 2000; Sabri et al., 2001). However, a more common approach includes accounting for these differences with neutral baselines.

In order to assess facilitative and interfering effects in the Stroop paradigm, a neutral baseline is necessary (Tzelgov et al., 1992; Neely and Kahan, 2001; Augustinova and Ferrand, 2012). For instance, Tzelgov et al. (1992) manipulated the proportion of neutral trials relative to congruent and incongruent color words, and found that a larger proportion of neutral words correlated with a larger proportion of interference comparing neutral and incongruent conditions, although facilitation was not impacted by the proportion of neutral trials. In consideration of the growing literature describing contingency effects and covariation in Stroop task design, it is important to ask what constitutes an appropriate neutral baseline (see Brown, 2011, for a recent discussion). We argue that in a context where covariation exists, a neutral baseline should also incorporate this covariation, as well as match the characteristics of the condition(s) it is being compared to (i.e., the congruent or incongruent condition). Thus, if there are more repetitions of congruent trials to equate the congruent and incongruent conditions, the neutral words should follow this covariation, whereby the congruent condition is only compared to a repeated neutral counterpart, and the incongruent condition is only compared to its less frequent neutral counterpart.

Dennis and Newstead (1981) provided a starting point in matching the contingency of neutral PHs with congruent and incongruent CWs and CWPHs, but omitted neutral words (e.g., “grief” serving as a counterpart to “green”), which does not allow for direct comparison of CWs and neutral baseline while matching on reading processes like whole-word recognition. In addition, contingency effects have not been studied with CA and CAPH congruency effects, where both congruent and incongruent stimuli are included. Color associate congruency effects are taken to reflect a purely semantic contribution (i.e., associates preclude the response level conflict that color words are vulnerable to), however, one must still control for a contingency learning confound.

In order to account for both congruency and contingency effects on facilitation and interference, we used two neutral baselines: one matched for the pair-frequency of congruent stimuli and one matched for the pair-frequency of incongruent stimuli. Experiment 1 utilized typical Stroop CWs (e.g., “blue”; matched neutral “blow”) and CWPHs (e.g., “bloo”; matched neutral “bloe”), whereas Experiment 2 utilized typical CAs (e.g., “ocean”; matched neutral “odor”) and CAPHs (e.g., “oshin”; matched neutral “ohder”). Experiments 3 and 4 were identical to Experiments 1 and 2, while also examining the effects of neutral baseline onset. For these first two experiments, we hypothesized that there should be significant Stroop effects over and above contingency effects (as assessed by the two neutral conditions). The extent of facilitation vs. inhibition will also be assessed, and we note although the extant literature points to greater inhibition than facilitation effects (e.g., Cheesman and Merikle, 1984; MacLeod, 1991; Tzelgov et al., 1992) these effects have never been compared before in the present design using color associates to isolate the semantic contribution to the Stroop effects.

## Experiment 1

### Methods

#### Participants

Twenty-five undergraduate students from the University of Saskatchewan received partial course credit for their participation. All were fluent speakers of English who reported normal or corrected-to-normal vision. The data from one participant was excluded from the analysis as the individual did not learn English as their first language, and was not familiar with all of the Standard English color names used in this experiment (i.e., red, orange, yellow, green, blue, purple, pink, white, gray). The experiment (and subsequent experiments) were approved by the University of Saskatchewan Research Ethics Board with written informed consent from all participants and performed in accordance with the Helsinki Declaration.

#### Stimuli

Following Anton et al. (2014), stimuli appeared in nine different font colors one-at-a-time, across four types of letter strings (CWs: e.g., “blue”; CWPHs: e.g., “bloo”; neutral words: e.g., “blow”; and neutral pseudohomophones: e.g., “bloe”; see Appendix in Supplementary Materials) in the center of a black screen. Stimuli were presented individually in bold, lowercase, 32-point Arial font. The longest stimulus, “perpull,” subtended a visual angle 2.864° in height × 10.570° in width. Each stimulus appeared in the following colors: red (E-Prime's red), orange (RGB: 210, 105, 0), yellow (E-Prime's yellow), green (E-Prime's green), blue (E-Prime's blue), purple (RGB: 159, 0, 159), pink (RGB: 255, 128, 192), white (E-Prime's white), and gray (RGB: 141, 141, 141). All font color and letter string pairings were used. The neutral letter strings were matched to the color letter strings for onset (same first phoneme), log word frequency HAL, length, number of orthographic neighbors, and number of phonographic neighbors [all *t*s_(8)_ ≤ 0.815, *p*s ≥ 0.439] based on values from the E-Lexicon database (http://elexicon.wustl.edu/; Balota et al., 2007). The pseudohomophones were generated to perfectly match their corresponding words in number of syllables and number of phonemes, see Anton et al. (2014).

The pairing of each letter string and font color was either congruent or incongruent. Stimuli were congruent if the font color matched what the letter string spelled (e.g., “blue” or “bloo” in blue font) and were incongruent if the two did not match (e.g., “blue” or “bloo” in orange font). Neutral stimuli were yoked to color-related stimuli in that they had a “congruent” color that they appeared in eight times and eight “incongruent” colors that they appeared in once (e.g., the letter strings “blue” and “blow” were presented eight times in the font color blue and once in each other color. For the purposes of referring to neutral conditions, the word congruent will be used to refer to any color/word combination that were paired frequently (i.e., eight times), and incongruent will be used to refer to color/word pairs that occurred infrequently (i.e., once), such that a congruent neutral refers to higher frequency color/word pairs and incongruent neutral refers to lower frequency color/word pairs. Thus, there were 72 congruent and 72 incongruent trials for each type of stimulus (color and neutral words), resulting in 576 trials in the entire experiment.

#### Apparatus and procedure

The stimuli were presented on a standard PC computer monitor through the use of E-Prime 2.0 software (Psychology Software Tools, Inc., http://www.pstnet.com), and vocal responses were obtained using a LabTec AM-22 microphone interfaced with the E-Prime voice key serial response box. Participants were tested individually in a quiet room. Participants were instructed to press the “Go” button on the manual response box to view each stimulus and to vocally name the font color of the stimulus as quickly and as accurately as possible, while ignoring what the letter string said.

Participants first completed a series of 18 practice trials, which were designed to familiarize them with the stimuli and allow the researcher to provide feedback about incorrect responses. They were then presented with two blocks of trials, one for words and one for pseudohomophones, with block order counterbalanced across participants. Within each block, stimulus presentation was random. In each trial, participants pressed the “Go” button to view the stimulus and named the font color of the letter string as quickly as possible and the experimenter coded their response. Participants did not receive feedback during the experiment and testing took ~20 min.

### Results

The response times (RTs) were measured in milliseconds (ms) and the median RT for each experimental condition was calculated for each participant's correct responses. The mean accuracy rate was also calculated for each participant by dividing the number of correct responses by the total number of unspoiled responses.

**Figure 1:**
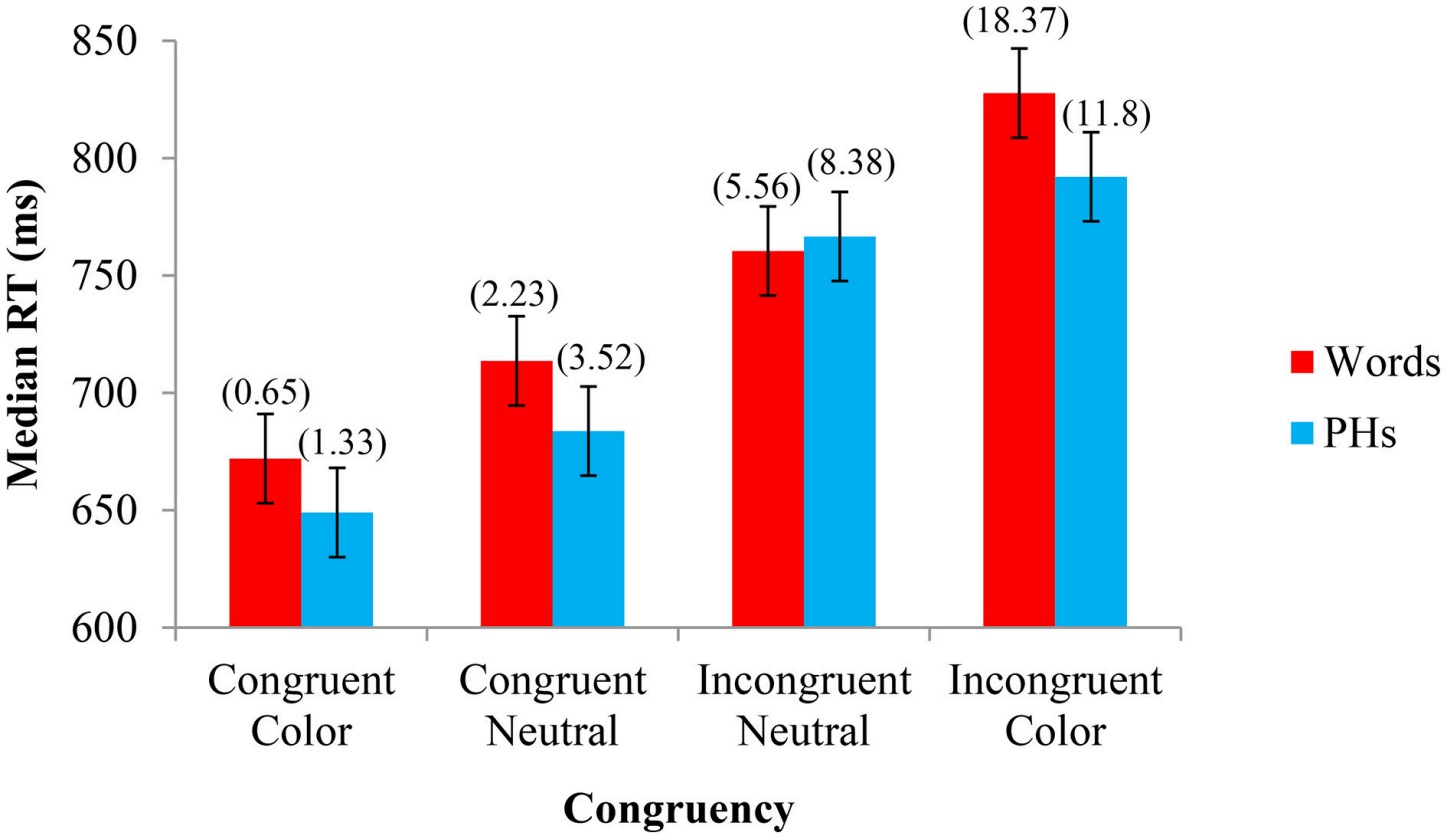
**Experiment 1—Color name stimuli: Median response time (RT) and percent errors (above) in naming the font color as a function of Letter String Type (word, PHs) and Congruency (congruent, neutral congruent, neutral incongruent, incongruent)**. The 95% confidence intervals (shown here by the error bars attached to each data point) indicated that, for both words and PHs, there were significant differences in response time between each specific congruency condition (i.e., the RTs for congruent color, congruent neutral, incongruent neutral, and incongruent color stimuli were significantly different from each other). The percent error confidence interval was ± 2.67%. Paired-samples *t*-tests support the 95% CIs for Experiment 1, whereby if the CIs reflected a significant difference, so too did the *t*-tests, all *t's*_(23)_ > 3.152, *p's* < 0.005.

The median RTs for each condition were compared using an omnibus 2 Letter String Type (word, PH) × 4 Congruency (congruent, neutral congruent, neutral incongruent, incongruent) repeated-measures general linear model (GLM) analysis of variance (ANOVA). As predicted, there was a main effect of Congruency (Congruent Color *M* = 660 ms, Congruent Neutral *M* = 699 ms, Incongruent Neutral *M* = 764 ms, Incongruent Color *M* = 810 ms), *F*_(1, 23)_ = 109.921, *MSE* = 1932.704, *p* < 0.001. There was no main effect of Letter String Type (Word *M* = 743 ms, PH *M* = 723 ms), *F*_(1, 23)_ = 2.698, *MSE* = 7550.225, *p* = 0.114, and therefore no significant difference between the RTs for pseudohomophones and words. There was also a significant interaction between Letter String Type and Congruency, *F*_(1, 23)_ = 5.484, *MSE* = 755.225, *p* = 0.002. The median RTs from each cell of the 2 × 4 ANOVA are shown in Figure 1. The 95% confidence intervals in this figure were calculated using the method of Loftus and Masson (1994). We also conducted a parallel analysis by-items (see Appendix in Supplementary Materials), where matched color and neutral words were analyzed within-items. There was a main effect of Congruency (Congruent Color *M* = 655 ms, Congruent Neutral *M* = 692 ms, Incongruent Neutral *M* = 750 ms, Incongruent Color *M* = 793 ms), *F*_(3, 24)_ = 73.93, *MSE* = 905, *p* < 0.001, and Letter String Type (Word *M* = 717 ms, PH *M* = 728 ms), *F*_(1, 8)_ = 11.073, *MSE* = 218.64, *p* = 0.010. The interaction was also significant between Letter String Type and Congruency, *F*_(3, 24)_ = 5.484, *MSE* = 285.464, *p* = 0.020, supporting the pattern of results by subjects, although the main effect of Letter String Type emerged significant.

The participants' percent errors were also analyzed in a 2 × 4 within-subjects GLM ANOVA. There was a main effect of Congruency (Congruent Color *M* = 0.010, Congruent Neutral *M* = 0.029, Incongruent Neutral *M* = 0.070, Incongruent Color *M* = 0.151), *F*_(1, 23)_ = 30.933, *MSE* = 0.006, *p* < 0.001, but no main effect of Letter String Type (Word *M* = 0.067, PH *M* = 0.063), *F*_(1, 23)_ = 0.218, *MSE* = 0.004, *p* = 0.645. As was found with the RT data, there was a significant interaction between Letter String Type and Congruency, *F*_(1, 23)_ = 12.336, *MSE* = 0.002, *p* < 0.001.

### Discussion

Experiment 1 investigated the separate contributions of congruency and contingency effects with CWs and CWPHs in the Stroop task. The faster responding to higher pair-frequency neutral stimuli reflects contingency learning, which occurred for both words and pseudohomophones. Although the difference between congruent and incongruent stimuli is typically shown as the Stroop effect, the difference between the pair-frequency matched neutral conditions reflects the proportion of the Stroop effect that can be accounted for by contingency learning. These results suggest that contingency learning can occur equally for PHs and actual words (similar to (Dennis and Newstead, 1981), although actual-word neutrals were omitted from their design), even though PHs require phonetic decoding to be meaningful. The genuine Stroop effect for CWs and CWPHs can also be seen above and beyond contingency effects. That is, relative to pair-frequency matched neutral words, both facilitation and interference were found with congruent and incongruent CWs and CWPHs, respectively. Overall, while CWs and CWPHs generate a genuine Stroop effect, a substantial portion of these significant effects (46/156 ms or ~30% for words, 83/143 ms or ~58% for PHs) is attributable to contingency learning in our experiment. In addition, examining the 95% confidence intervals of Experiment 1 (see Figure 1), our results also showed that incongruent CWs showed more interference than incongruent CWPHs relative to their respective neutral baselines, whereas congruent CWs did not show more facilitation than congruent CWPHs, which paralleled the findings of Dennis and Newstead (1981).

The results of Experiment 1 support the notion that contingency learning occurs in the Stroop task. However, color name stimuli do not address the contribution of semantic processing as such effects can reflect response competition. Given that Experiment 1 addresses the main question of what amount of interference and facilitation remains in the Stroop task when controlling for contingency effects, this design also lends itself to addressing the parallel question for color associate stimuli, which allows for the isolation of the semantic component of the Stroop effect. Although the Stroop effects found with color associate stimuli are assumed to reflect semantic processing (e.g., Klein, 1964; Augustinova and Ferrand, 2012; Anton et al., 2014), their effects in the classic design Stroop task (employing all combinations of font colors and words) may conflate semantic and contingency effects. Thus, Experiment 2 aimed to estimate the magnitude of semantic Stroop interference and facilitation in font color naming for CAs and CAPHs when contingency effects are controlled using pair-frequency matched neutral words.

## Experiment 2

### Methods

#### Participants

Twenty-four additional undergraduate students from the University of Saskatchewan were recruited. These students were undergraduate students who either received partial course credit in introductory psychology or received a monetary compensation of five dollars for their participation. All were fluent speakers of English.

#### Stimuli

Color associate words (e.g., “ocean”; from (Anton et al., 2014), see Appendix in Supplementary Materials) were considered congruent if the font color matched the color of the associate word's referent (e.g., “ocean” in blue font), and incongruent if they did not match (e.g., “ocean” in orange font). A new set of neutral items was matched to the color associates for onset, log frequency HAL, length, number of orthographic neighbors, and number of phonographic neighbors [all *t*s_(8)_ ≤ 1.322, *p*s ≥ 0.223] based on values from the E-Lexicon database (http://elexicon.wustl.edu/; Balota et al., 2007). As in Experiment 1, the congruency of the neutral items was determined on the basis of the frequency with which the color-related letter strings and the font colors were paired. All other aspects were identical to Experiment 1.

#### Apparatus and procedure

The apparatus and procedure were identical to those used in Experiment 1.

### Results

The same analyses of the data performed in Experiment 1 were performed in Experiment 2. The median RTs from each experimental condition were analyzed with a 2 Letter String Type (word, PH) × 4 Congruency (congruent, neutral congruent, neutral incongruent, incongruent) repeated-measures GLM ANOVA. As in Experiment 1, there was a main effect of Congruency (Congruent Associate *M* = 662 ms, Congruent Neutral *M* = 658 ms, Incongruent Neutral *M* = 686 ms, Incongruent Associate *M* = 685 ms), *F*_(1, 23)_ = 20.600, *MSE* = 523.652, *p* < 0.001, but no main effect of Letter String Type (Word *M* = 676 ms, PH *M* = 670 ms), *F*_(1, 23)_ = 1.543, *MSE* = 1360.709, *p* = 0.227. Unlike the first experiment, however, there was no significant interaction between Congruency and Letter String Type, *F*_(1, 23)_ = 0.527, *MSE* = 398.309, *p* = 0.665. We also conducted a parallel analysis by-items (see Appendix in Supplementary Materials), where matched color and neutral words were analyzed within-items. The main effects of Congruency (Congruent Associate *M* = 649 ms, Congruent Neutral *M* = 645 ms, Incongruent Neutral *M* = 672 ms, Incongruent Associate *M* = 673 ms), *F*_(3, 24)_ = 5.303, *MSE* = 731.284, *p* = 0.006, and Letter String Type (Word *M* = 665 ms, PH *M* = 654 ms), *F*_(1, 8)_ = 6.857, *MSE* = 301.78, *p* = 0.031, were significant. The interaction between Letter String Type and Congruency was not significant, *F*_(3, 24)_ = 1.018, *MSE* = 126.225, *p* = 0.402. These results show largely the same pattern as by-subjects, although the main effect of Letter String Type emerged significant.

**Figure 2:**
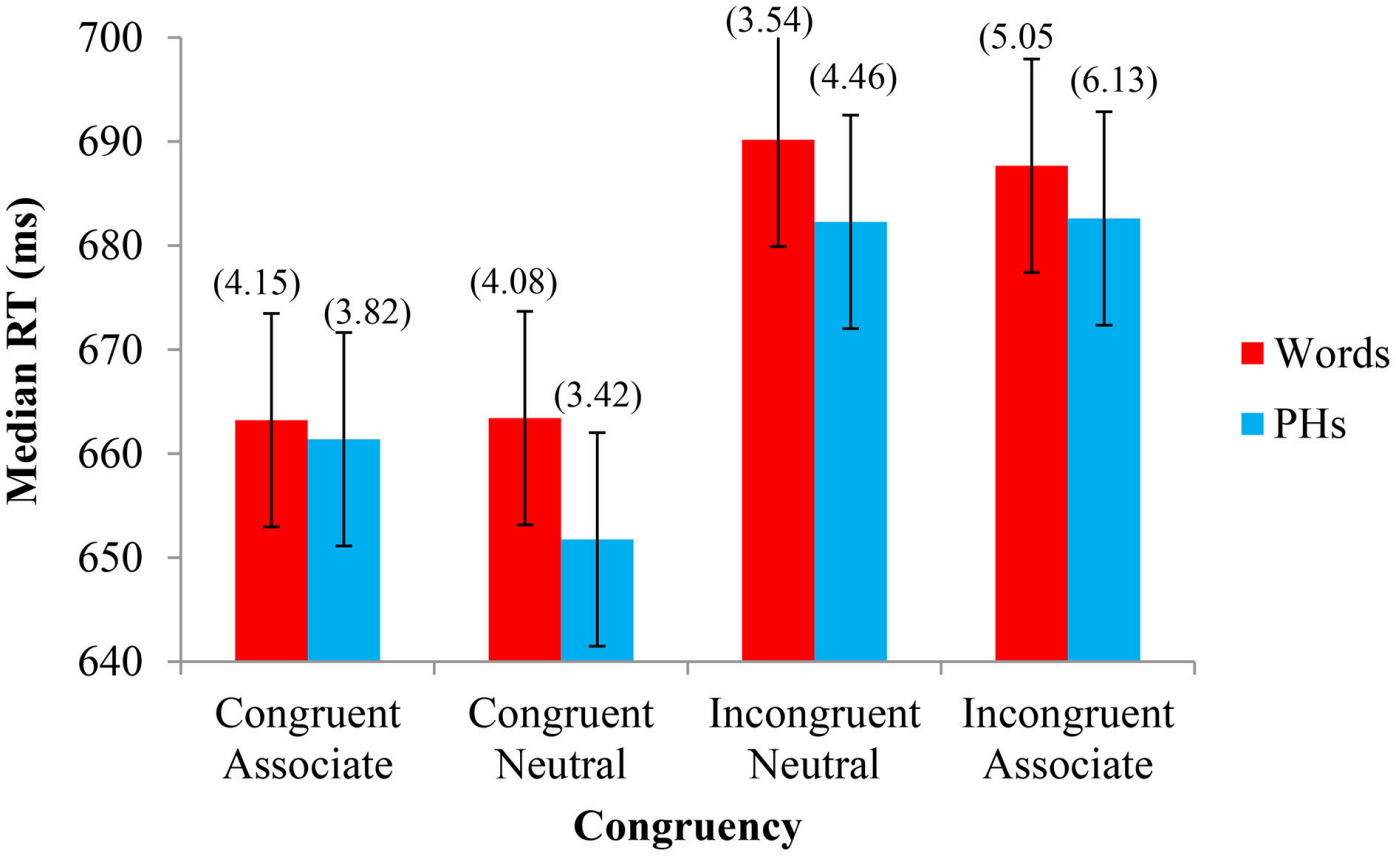
**Experiment 2—Color associated stimuli: Median response time (RT) and percent errors (above) in naming the font color as a function of Letter String Type (word, PH) and Congruency (congruent, neutral congruent, neutral incongruent, incongruent)**. The 95% confidence intervals (shown here for RT by the error bars attached to each data point) indicate that, for word stimuli, there is no difference in the time taken to respond to congruent color words and congruent neutral words and there is also no difference between incongruent color and incongruent neutral words. For PH stimuli, there is no difference in RTs between congruent neutral and congruent color words, or between incongruent neutral and incongruent color words. The percent error confidence interval was ± 1.34%. Paired-samples *t*-tests support the 95% CIs for Experiment 2, whereby if the CIs reflected a significant difference, so too did the *t*-tests, all *t's*_(23)_ > 3.815, *p's* < 0.002.

Percent errors were also analyzed in a 2 × 4 GLM ANOVA. There was a main effect of Congruency (Congruent Associate *M* = 0.039, Congruent Neutral *M* = 0.038, Incongruent Neutral *M* = 0.040, Incongruent Associate *M* = 0.056), *F*_(1, 23)_ = 4.332, *MSE* = 0.001, *p* = 0.007, but no main effect of Letter String Type (Word *M* = 0.042, PH *M* = 0.046), *F*_(1, 23)_ = 0.159, *MSE* = 0.002, *p* = 0.694. As with the RT data, there was no significant interaction between Congruency and Letter String Type, *F*_(1, 23)_ = 1.226, *MSE* = 0.001, *p* = 0.307. The median RTs and percent errors for each condition are shown in Figure 2.

### Discussion

Experiment 2 investigated the separate contributions of congruency and contingency effects with CAs and CAPHs in the Stroop task. Similar to Experiment 1, faster responding occurred with higher pair-frequency neutral stimuli for both words and PHs, which is attributable to contingency learning. In contrast to Experiment 1, however, relative to pair-frequency matched neutral words, no significant facilitation was found with congruent CAs and CAPHs and no significant interference was found with CAs and CAPHs. Given that all of the color associate Stroop effect can be attributed to contingency learning effects (i.e., 27 ms neutral/25 ms Stroop for words and 30 ms neutral/22 ms Stroop for PHs), there is clear evidence to suggest that pair-frequency contingency learning is responsible for the Stroop effect with these color associated stimuli, at least for the proportions of congruency and contingency used here.

It is important to note, however, that in most studies employing CAs, congruent stimuli are often omitted from the design (e.g., Klein, 1964; Augustinova and Ferrand, 2012), and have demonstrated interference with incongruent stimuli relative to pair-frequency matched neutral words. A possible explanation for why Experiment 2 did not demonstrate the same interference may involve inclusion of a congruent condition, although this appears unlikely. Specifically, the inclusion of congruent stimuli, and greater proportions of congruent stimuli relative to incongruent stimuli, generally increase the overall size of the Stroop effect (Cheesman and Merikle, 1986; Lorentz et al., 2015).

A more likely explanation may relate to the onset matching of neutral words. Learning that the neutral stimuli's onsets match the congruent and incongruent stimuli may cue participants to an association between stimuli like “odor” and “ocean.” In this way, onset matching may mimic the effect of contingency learning and mask genuine semantic effects with CAs and CAPHs. If this were the case, neutral words would take on the related color attributes of their associate counterparts, and therefore, show the similar patterns of responding in RT and percent errors. While onset matching is common (Hintzman et al., 1972; Dennis and Newstead, 1981), the impact of such matching is not clear in relation to contingency learning, especially in assessing facilitation and interference. Although contingency learning is shown with lists of neutral words (Schmidt et al., 2007) and lists of color words (Bugg and Hutchison, 2013), the effects of matching lists of neutral words and color words for onset and contingency remains an open question.

In the following two experiments, we addressed this gap in the literature to examine how using neutral words without onset matching will change the pattern of contingency and congruency effects found in Experiments 1 and 2. We hypothesized that, if onset matching created an association between neutral and color-related words, then using neutral stimuli that do not have matched onsets to the congruent stimuli would reveal a greater difference between color-related words and their corresponding neutral baselines. This may present as larger facilitation and interference and smaller differences in contingency learning, as shown by the difference in pair-frequency neutral baselines.

## Experiment 3

### Methods

#### Participants

Twenty-four additional undergraduate students from the University of Saskatchewan were recruited. These students were undergraduate students who received partial course credit in their introductory psychology course for their participation. All were fluent speakers of English and all reported normal or corrected-to-normal vision.

#### Stimuli

The same nine color words that were used in Experiment 1 (see Appendix in Supplementary Materials) served as the stimuli for Experiment 3. A new set of neutral items was matched to the color words for log frequency HAL, length, number of orthographic neighbors, and number of phonographic neighbors [all *t*s_(8)_ ≤ 0.287, *p*s ≥ 0.781] based on values from the E-Lexicon database (http://elexicon.wustl.edu/; Balota et al., 2007). As in Experiment 1, the congruency of the neutral items was determined on the basis of the frequency with which the letter string and the font color were paired. Unlike Experiment 1, however, the neutral stimuli did not share their onset with any of the color word stimuli. All other aspects of the stimuli were identical to Experiment 1.

#### Apparatus and procedure

The apparatus and procedure were identical to those used in the first two experiments.

### Results

The same analyses of the data performed in the first two experiments were performed in Experiment 3. The median RTs from each experimental condition were analyzed with a 2 Letter String Type (word, PH) × 4 Congruency (congruent, neutral congruent, neutral incongruent, incongruent) repeated-measures GLM ANOVA. As in the first two experiments, there was a main effect of Congruency (Congruent Color *M* = 671 ms, Congruent Neutral *M* = 742 ms, Incongruent Neutral *M* = 749 ms, Incongruent Color *M* = 829 ms), *F*_(1, 23)_ = 108.982, *MSE* = 1852.439, *p* < 0.001, but no main effect of Letter String Type (Word *M* = 755 ms, PH *M* = 740 ms), *F*_(1, 23)_ = 1.390, *MSE* = 7462.320, *p* = 0.251. There was no significant interaction between Congruency and Letter String Type, although this interaction approached significance, *F*_(1, 23)_ = 2.632, *MSE* = 830.390, *p* = 0.057. We also conducted a parallel analysis by-items (see Appendix in Supplementary Materials), where matched color and neutral words were analyzed within-items. The main effects of Congruency (Congruent Color *M* = 673 ms, Congruent Neutral *M* = 738 ms, Incongruent Neutral *M* = 749 ms, Incongruent Color *M* = 827 ms), *F*_(3, 24)_ = 61.327, *MSE* = 1162.769, *p* < 0.001, and Letter String Type (Word *M* = 751 ms, PH *M* = 742 ms), *F*_(1, 8)_ = 6.029, *MSE* = 242.574, *p* = 0.040, were significant. The interaction between Letter String Type and Congruency was not significant, *F*_(3, 24)_ = 1.438, *MSE* = 225.744, *p* = 0.256. These results show largely the same pattern as by-subjects, although the main effect of Letter String Type emerged significant.

**Figure 3:**
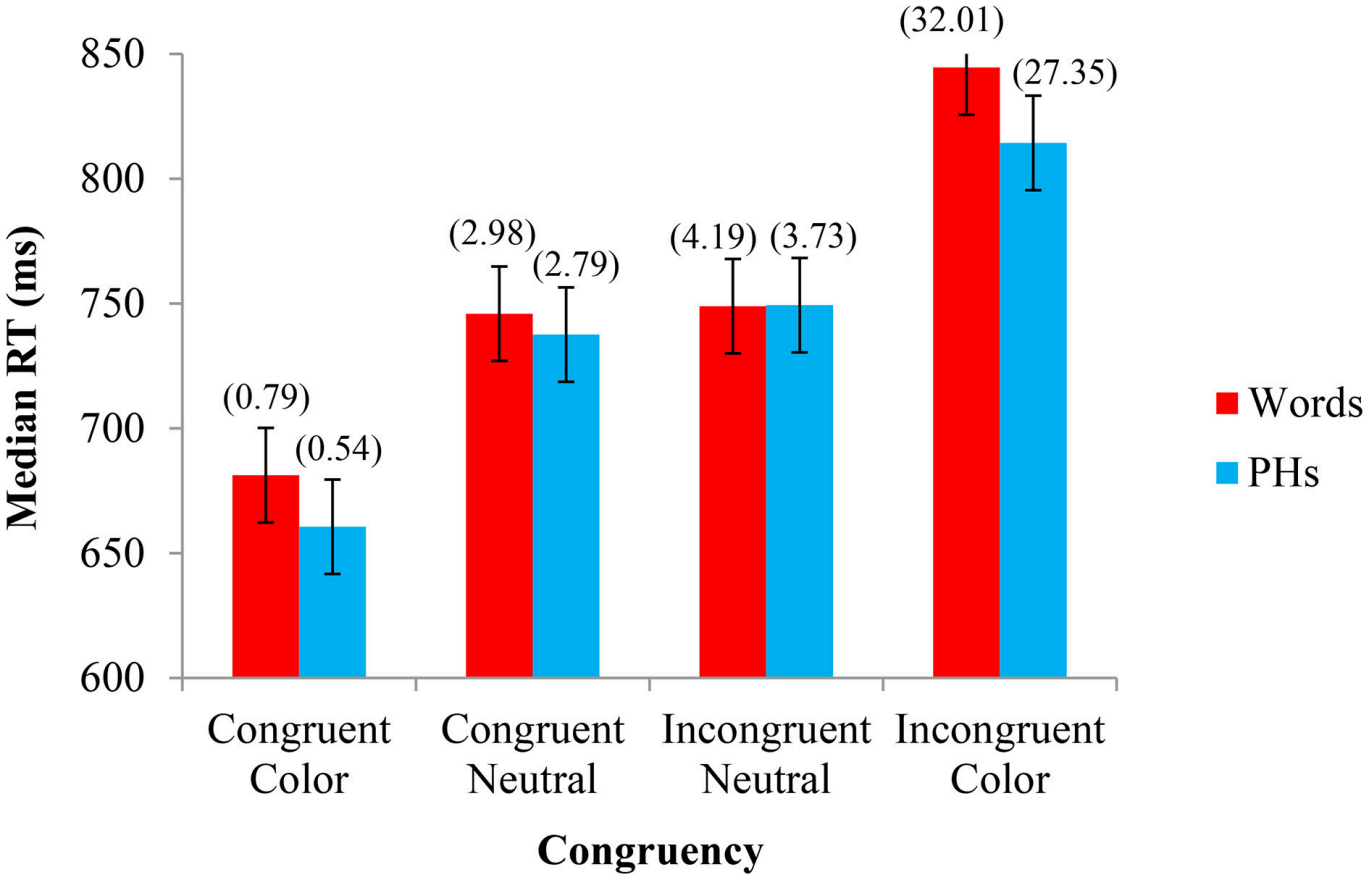
**Experiment 3—Color name stimuli: Median response time (RT) and percent errors (above) in naming the font color as a function of Letter String Type (word, PH) and Congruency (congruent, neutral congruent, neutral incongruent, incongruent)**. The 95% confidence intervals (shown here for RT by the error bars attached to each data point) indicate that, for both word and PH stimuli, there is no difference in the time taken to respond to the congruent neutral words and the incongruent neutral words. There is, however, a significant difference between the congruent and congruent neutral conditions (congruent words responded to faster) and between the incongruent neutral and incongruent conditions (incongruent words responded to slower) for both words and PHs. The percent error confidence interval was ± 1.97%. Paired-samples *t*-tests support the 95% CIs for Experiment 3, whereby if the CIs reflected a significant difference, so too did the *t*-tests, all *t's*_(23)_ > 6.598, *p's* < 0.001.

Percent errors were also analyzed in a 2 × 4 GLM ANOVA. There was a main effect of Congruency (Congruent Color *M* = 0.007, Congruent Neutral *M* = 0.029, Incongruent Neutral *M* = 0.040, Incongruent Color *M* = 0.297), *F*_(1, 23)_ = 213.120, *MSE* = 0.004, *p* < 0.001, a main effect of Letter String Type (Word *M* = 0.1, PH *M* = 0.086), *F*_(1, 23)_ = 7.306, *MSE* = 0.001, *p* = 0.013, and a significant interaction between Congruency and Letter String Type, *F*_(1, 23)_ = 5.934, *MSE* = 0.001, *p* = 0.001. The median RTs and percent errors for each condition are shown in Figure 3.

### Discussion

Experiment 3 investigated the separate contributions of congruency and contingency effects with CWs and CWPHs in the Stroop task by employing neutral words *not* matched on onset. Similar to Experiment 1, a genuine Stroop effect was found for CWs and CWPHs above and beyond contingency effects, where both facilitation and interference were found with congruent and incongruent CWs and CWPHs relative to their neutral counterparts, respectively. In contrast to Experiment 1, though, contingency effects were quite minimal in the neutral conditions when forgoing onset matched neutral words and PHs (11/154 ms or ~7% for CWs, 11/80 ms or ~14% CWPHs), and the proportion of genuine Stroop effect was much larger. Together with Experiment 1, these results bring strong support for an onset effect, whereby participants learned an association between neutral and color related words when onset matching was employed, and minimized when they were not matched.

As we found a significant reduction in the contribution of contingency effects in this task compared to Experiment 1, this design also lends itself to addressing the parallel question for CAs and CAPHs. Experiment 2 found that the genuine Stroop effect was altogether accounted for by contingency learning when employing first-letter matched neutral stimuli. However, as Experiment 3 found a significant reduction in contingency learning when eliminating onset matching (i.e., using “red” vs. “fun” instead of “red” vs. “rap”), suggesting that when participants have neutral words that do not match the onset of color related stimuli, there is better control over the spread of association in the semantic network, and thus no inflation of the contingency effect. In other words, using neutral stimuli that do not match onset to the color related stimuli, the onset effect does not contribute to the contingency effect. We predicted a genuine Stroop effect for CAs and CAPHs should occur when omitting onset matching in our neutral baselines. Thus, Experiment 4 aimed to extend Experiment 2 in estimating the magnitude of Stroop interference and facilitation in font color naming due to CAs and CAPHs when contingency effects are controlled using pair-frequency matched neutral words that do not share the same onset as the associate stimuli.

## Experiment 4

### Methods

#### Participants

Twenty-four additional undergraduate students from the University of Saskatchewan were recruited. These students were undergraduate students who received partial course credit in their introductory psychology course for their participation. All were fluent speakers of English and all reported normal or corrected-to-normal vision.

#### Stimuli

The same nine color associate words that were used in Experiment 2 (see Appendix in Supplementary Materials) served as the stimuli for Experiment 4. A new set of neutral items was matched to the color words for log frequency HAL, length, number of orthographic neighbors, and number of phonographic neighbors [all *t*s_(8)_ ≤ 1.000, *p*s ≥ 0.347] based on values from the E-Lexicon database (http://elexicon.wustl.edu/; Balota et al., 2007). Importantly, in contrast to Experiment 2, the neutral stimuli did not share their onset with any of the color associate stimuli (including the color associate to which they were specifically matched). As in the previous experiments, the congruency of the neutral items was determined on the basis of the frequency with which the letters string and the font color were paired. All other aspects of this experiment were identical to Experiment 2.

#### Apparatus and procedure

The apparatus and procedure were identical to those used in the first three experiments.

### Results

The same analyses of the data performed in the first three experiments were performed in Experiment 4. The median RTs from each experimental condition were analyzed with a 2 Letter String Type (word, PH) × 4 Congruency (congruent, neutral congruent, neutral incongruent, incongruent) repeated-measures GLM ANOVA. As with all previous experiments, there was a main effect of Congruency (Congruent Associate *M* = 755 ms, Congruent Neutral *M* = 767 ms, Incongruent Neutral *M* = 774 ms, Incongruent Associate *M* = 779 ms), *F*_(1, 23)_ = 14.663, *MSE* = 363.381, *p* < 0.001. There was no main effect of Letter String Type (Word *M* = 769 ms, PH *M* = 769 ms), *F*_(1, 23)_ < 0.001, *MSE* = 4486.293, *p* = 0.993, but there was a significant interaction between Congruency and Letter String Type, *F*_(1, 23)_ = 3.263, *MSE* = 490.739, *p* = 0.027. We also conducted a parallel analysis by-items, where matched color and neutral words were analyzed within-items. The main effect of Letter String Type was significant (Word *M* = 765 ms, PH *M* = 758 ms), *F*_(1, 8)_ = 6.342, *MSE* = 126.141, *p* = 0.036, although the main effect of Congruency was not significant (Congruent Associate *M* = 750 ms, Congruent Neutral *M* = 760 ms, Incongruent Neutral *M* = 765 ms, Incongruent Associate *M* = 771 ms), *F*_(3, 24)_ = 1.719, *MSE* = 809.412, *p* = 0.190. Similar to the by-subjects analysis, the interaction between Congruency and Letter String Type showed a trend toward interaction, *F*_(3, 24)_ = 2.522, *MSE* = 106.723, *p* = 0.082, showing largely the same pattern by-items as by-subjects.

**Figure 4:**
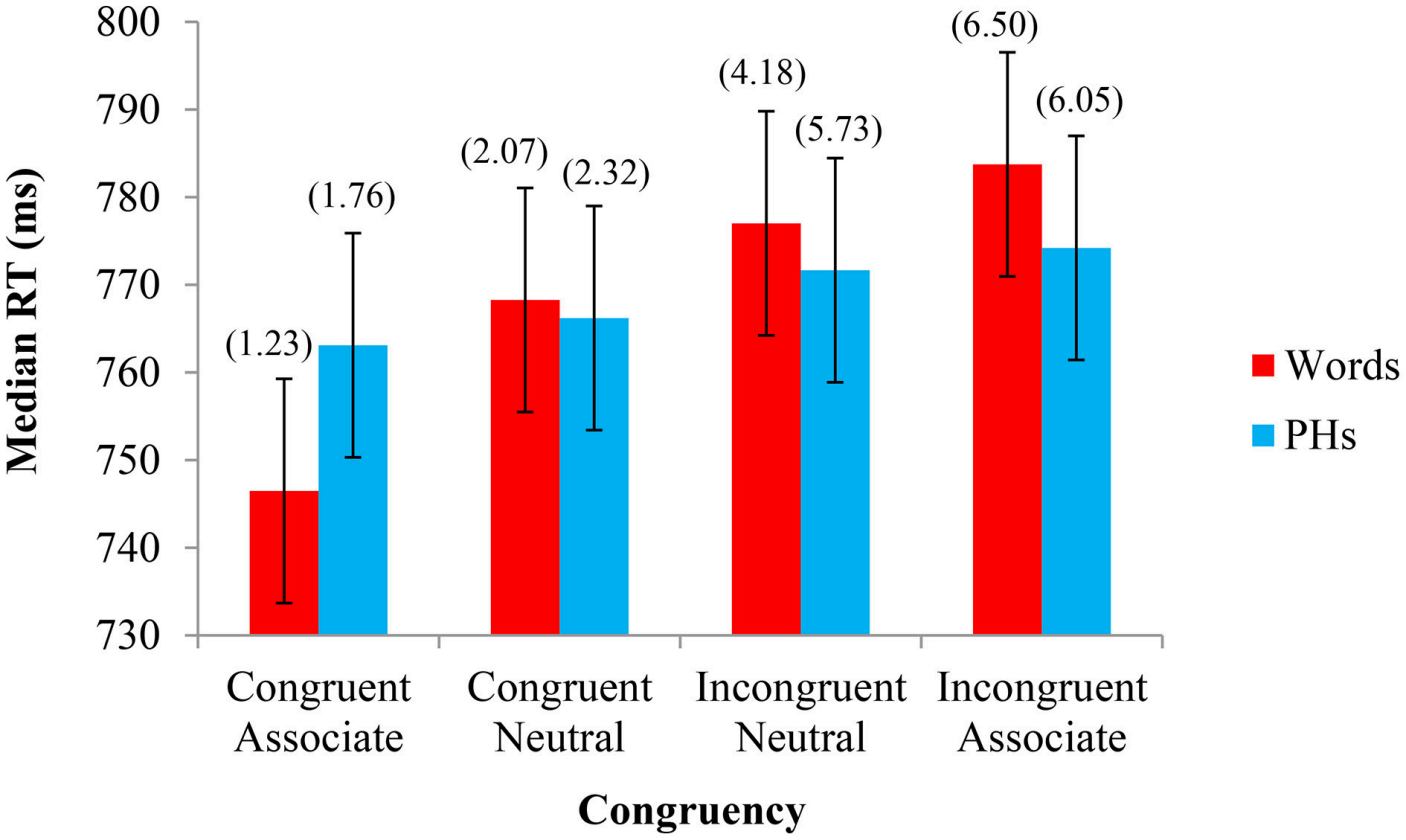
**Experiment 4—Color associated stimuli: Median response time (RT) and percent errors (above) in naming the font color as a function of Letter String Type (word, PH) and Congruency (congruent, neutral congruent, neutral incongruent, incongruent)**. The 95% confidence intervals (shown here for RT by the error bars attached to each data point) indicate that, for PHs there was no significant difference in the time taken to respond to stimuli across any of the four congruency conditions. There was no difference in responding time, however, between the two neutral conditions, or between the incongruent neutral and incongruent associate conditions. The percent error confidence interval was ± 0.979%. Paired-samples *t*-tests support the 95% CIs for Experiment 1, whereby if the CIs reflected a significant difference, so too did the *t*-tests, all *t's*_(23)_ > 2.443, *p's* < 0.024. Supporting the critical difference for words between Congruent Associate and Congruent Neutral conditions, a paired-samples *t*-test was also significant, t_(23)_ = 3.31, *p* = 0.003. Also supporting this effect with a Bayesian Analysis, where prior odds were set to 1 for both null and alternative hypotheses, the Bayes factor of 11.98 constituted a posterior probability of 0.923 for the alternative hypothesis, and thus strong evidence for this facilitation effect.

Percent errors were also analyzed in a 2 × 4 GLM ANOVA. There was a main effect of Congruency (Congruent Associate *M* = 0.015, Congruent Neutral *M* = 0.022, Incongruent Neutral *M* = 0.050, Incongruent Associate *M* = 0.063), *F*_(1, 23)_ = 41.333, *MSE* = 0.001, *p* < 0.001, no main effect of Letter String Type (Word *M* = 0.035, PH *M* = 0.040), *F*_(1, 23)_ = 1.109, *MSE* = 0.001, *p* = 0.303, and no significant interaction between Congruency and Letter String Type, *F*_(1, 23)_ = 1.802, *MSE* < 0.001, *p* = 0.155. The median RTs and percent errors for each condition are shown in Figure 4.

### Discussion

Experiment 4 investigated the separate contributions of congruency and contingency effects with CAs and CAPHs in the Stroop task, while using neutral words and PHs that were purposely not matched to the onsets of the color associate stimuli. We found a facilitation effect for CA words relative to their neutral counterparts, an effect that did not reach significance when pair-frequency matched neutral baselines were matched to the onsets of the color associate stimuli in Experiment 2, but otherwise no other genuine facilitation or interference effects reached significance. Similarly to Experiment 3, the difference between pair-frequency matched neutral baselines did not reach significance, supporting the hypothesis that onset matching enhances learning the association of color words with neutral words, as shown in Experiments 1 and 2. A comparison of congruent and incongruent color-related PHs did not reveal a congruency effect (e.g., Anton et al., 2014), nor a contingency effect, suggesting that the addition of neutral words may have diluted the effect in the present design (~11 vs. ~25 ms in Anton et al., 2014).

## General discussion

Our experiments investigated the relative contribution of facilitation and interference in the Stroop effect using two neutral baselines, each matching the pair-frequency of congruent and incongruent words. Using this design, we could evaluate the effect of contingency (the frequencies with which colors and words were paired) apart from genuine Stroop effects (the amount of facilitation and interference relative to baseline). This design was employed with CWs and CAs, as well as their PH counterparts to evaluate how contingency and genuine Stroop effects may differ between the letter string types. Finally, we investigated how the use of onset matched and onset unmatched neutral letter strings affected genuine Stroop and contingency effects.

The current experiments point to two major issues concerning facilitation and interference in the Stroop task. It is clear that a neutral baseline must be used to describe the relative contributions of facilitation or interference (e.g., Roelofs, 2010), but a single neutral baseline may not be appropriate when there are more incongruent color/word pairings than congruent color/word pairings, an artifact of Stroop designs with greater than two colors and words (Schmidt and Besner, 2008). When employing two neutral baselines, matched to the congruent and incongruent color/word pairing frequencies, our results suggest that contingency effects are robust in the Stroop task depending on the neutral words being used. In the presence of onset matched neutral words, participants show contingency effects more strongly than with unmatched onset stimuli. Contingency effects failed to reach significance in the absence of onset matched neutral words across two experiments. Previous research indicates that contingency learning can occur with color-naming in a set of four neutral words (Schmidt et al., 2007), but perhaps this is diluted or even eliminated by including both color-related and neutral stimuli in a larger set as used here, especially when onsets in neutral stimuli are not matched to their color associated counterparts. These effects provide a starting point for establishing the boundary conditions of contingency learning in regards to the size of the set, as well as the effect of onset matching between stimulus types.

Our experiments also examined the difference between CWs, CAs, and their PH counterparts while also comparing onset matching of neutral to color related stimuli. When matching these onsets, we find strong support for contingency effects with CWs, CWPHs, CAs, and CAPHs when compared to pair-frequency matched neutral trials, both by-subjects and by-items (see Appendix in Supplementary Materials). Color associate words showed significant facilitation, whereas only CWs and CWPHs generated significant facilitation and interference, over and above contingency effects. As CWs and CWPHs share the pronunciation of responses, in addition to sharing semantic representations with the font colors presented, the magnitude of interference and facilitation they produce relative to neutral trials may be due to response competition, semantic competition, or both (e.g., Dennis and Newstead, 1981; Anton et al., 2014). In other words, the current results complement prior studies in demonstrating additional response competition with CWs and CWPHs (compared to CAs and CAPHs) in generating significant interference and facilitation relative to their neutral word counterparts, respectively.

Color associated stimuli, on the other hand, share only semantic representations with the font colors presented in the Stroop task, and so their facilitation or interference has been taken to reflect semantic contributions (e.g., Klein, 1964; Augustinova and Ferrand, 2012; Anton et al., 2014). In terms of semantic influence, as measured by color associate Stroop effects, we show a clear facilitation-only effect on RT for color associate words, suggesting that there is at least some degree of automatic semantic access for such words. In addition, error rates point to an interference effect of CAs, suggesting that an interference effect may also be found with RTs if the design could include greater emphasis on color related stimuli. An interesting future experiment would include a larger proportion of color related stimuli by repeating these blocks while keeping the pair frequency of neutral stimuli the same, to explore how increasing the proportion of color related stimuli enhances Stroop effects.

Semantically driven effects have been shown under other conditions. For instance, Augustinova and Ferrand (2012) used a 2/3 incongruent to 1/3 neutral proportion and obtained significant interference, and Dalrymple-Alford (1972) employed 1/8 congruent, 3/8 incongruent, and 4/8 neutral proportion, showing both facilitation and interference. An important consequence of this variability is that evaluating the contributions of contingency learning, or differences in covariation, apart from semantic interference is left uncertain. In equating the neutral baselines for contingency, our design also evaluates the relative amounts of facilitation and interference, which have largely shown greater interference than facilitation in MacLeod's (1991) review of the literature. Color words and their PHs, when neutral baselines are matched for onset, show relatively similar effects, with 39 ms of facilitation and 46 ms of interference. When onset is not matched with baselines, color words and their PHs show 65 ms of facilitation and 60 ms of inhibition, also suggesting nearly similar sized effects. When evaluating the semantic contribution using color associates, the matched neutral basline condition shows no significant facilitation or interference. However, with color associate words and unmatched neutral baselines, which is arguably the more appropriate neutral baseline, our results show a facilitation-dominant effect when controlling for contingency. Thus, our present findings bring evidence for both facilitation and interference with CWs, and strong evidence for facilitation dominance with CAs.

Importantly, the present experiments have answered some questions concerning the basis of semantically driven Stroop effects, and provided a new approach to separate the effects of facilitation and interference from contingency using two neutral baselines. These baselines indicated that participants learn an association of color with neutral words in the presence of matching onsets, and that contingency effects are not a simple story of repetition. Clearly, contingency effects interact with neutral onset matching, showing larger effects of contingency when the neutral onsets match their corresponding color words or associate stimuli. In fact, the genuine Stroop effect found with CAs was detected only when neutral onsets were not matched, and thus are not associated by first letter to the target representations in the semantic network. As such, the present findings have isolated the semantic contribution of CAs to color naming in the Stroop task, and provided a new method for exploring both facilitation and interference when the task requires the integration of multiple sources of information.

## Conclusion

Although compelling in its simplicity, Stroop effects reflect more than congruency between color and word. In fact, there is an influence of both context in terms of the neutral baselines included, and pair-frequency learning that modulates the effects of color-words, color associates, and their PH counterparts. Even the choice of first-letter for neutral stimuli can alter the interpretation of facilitation and interference influencing the overall Stroop effect. In order to account for these factors, the appropriate pair-frequency neutral baselines described here provide a basis for elucidating the mechanisms of facilitation and interference, while controlling for artifacts and inherent design biases of the Stroop paradigm. We believe that there is great potential in the two-neutral baseline design for elucidating genuine facilitation and inhibition from contingency effects. Our initial exploration using this paradigm has demonstrated that appropriate pair-frequency matched neutral baselines result in significant genuine facilitation and interference on RT for CWs and CWPHs, and facilitation at the semantic level for color associate word stimuli, and thus supports the notion of automatic processing of words through to the semantic level. The implications for models of reading are clear in that all models would need to implement different degrees of automatic semantic processing between lexical and sub-lexical pathways, which are not implemented in any current models. Specifically, we have shown significant automatic semantic processing for lexical, but not sub-lexical stimuli, in the present design. It will be critical in further exploring these influences to continue to employ the appropriate neutral baselines to account for the contextual impact of contingencies and phonemic onset.

## Author note

Department of Psychology, 9 Campus Dr., University of Saskatchewan, Saskatoon, Saskatchewan, S7N 5A5, Canada. This research was supported by Natural Science and Engineering Research Council of Canada discovery grants to the senior author, RB (182968-2013–2018) and Undergraduate Student Research Awards to authors CE, TM, and KA, as well as Canadian Graduate Scholarships to LG, CE, and KA and a Saskatchewan Innovation and Opportunity Scholarship to EL. Correspondence to RB, phone: (306) 966-6679, fax: (306) 966-6630, email: ron.borowsky@usask.ca.

## Author contributions

EL designed experiments, conducted and reported analyses, drafted the main document. TM Conducted experiments, compiled, and analyzed data, constructed figures, wrote methods, edited main document. CE designed experiments, analyzed data, and edited main document. LG provided ideas, insights, and content to discussion, edited main document. KA designed experiments, conducted experiments, analyzed, and reported data. RB designed experiments, supervised the work, and edited main document

### Conflict of interest statement

The authors declare that the research was conducted in the absence of any commercial or financial relationships that could be construed as a potential conflict of interest.
