# Supplementary material for: Disentangling Genuine Semantic Stroop Effects in Reading from Contingency Effects: On the Need for Two Neutral Baselines
Source: Front Psychol. 2016 Mar 17;7:386. doi: 10.3389/fpsyg.2016.00386 (PMC4794500; doi:10.3389/fpsyg.2016.00386)
Supplement: Supplementary file 1 [file DataSheet1.docx]

**Appendix: Lexical Characteristics of Word and PH Stimuli**

| Word Stimulus | Length | Log Frequency HAL | Orthographic N | Phonographic N | Congruent RT | | Incongruent RT | |
| --- | --- | --- | --- | --- | --- | --- | --- | --- |
|  |  |  |  |  | M | SD | M | SD |
| **Experiment 1** |  |  |  |  |  |  |  |  |
| Color Words |  |  |  |  |  |  |  |  |
| red | 3 | 11.55 | 11 | 10 | 635 | 163 | 828 | 251 |
| orange | 6 | 9.51 | 1 | 0 | 643 | 145 | 834 | 184 |
| yellow | 6 | 9.87 | 3 | 3 | 660 | 184 | 802 | 283 |
| green | 5 | 11.42 | 4 | 4 | 687 | 171 | 798 | 249 |
| blue | 4 | 11.40 | 4 | 4 | 667 | 186 | 811 | 300 |
| purple | 6 | 9.15 | 0 | 0 | 659 | 188 | 791 | 260 |
| pink | 4 | 9.48 | 14 | 8 | 693 | 201 | 805 | 333 |
| white | 5 | 11.92 | 3 | 2 | 637 | 170 | 785 | 239 |
| gray | 4 | 9.45 | 8 | 4 | 668 | 183 | 794 | 287 |
| Mean | 4.78 | 10.42 | 9.00 | 3.89 | **661** | 177 | **805** | 265 |
|  | | |  |  |  |  |  |  |
| Neutral Words |  |  |  |  |  |  |  |  |
| rap | 3 | 8.58 | 19 | 16 | 659 | 157 | 760 | 223 |
| office | 6 | 11.39 | 0 | 0 | 724 | 170 | 737 | 273 |
| yearly | 6 | 7.95 | 3 | 2 | 724 | 181 | 750 | 370 |
| great | 5 | 12.47 | 3 | 2 | 720 | 228 | 722 | 236 |
| blow | 4 | 9.74 | 8 | 4 | 689 | 274 | 754 | 213 |
| puppet | 6 | 8.30 | 0 | 0 | 730 | 261 | 771 | 219 |
| pack | 4 | 10.32 | 16 | 13 | 706 | 183 | 745 | 213 |
| which | 5 | 13.80 | 0 | 0 | 655 | 132 | 734 | 178 |
| grab | 4 | 9.23 | 7 | 5 | 713 | 186 | 733 | 693 |
| Mean | 4.78 | 10.20 | 6.22 | 4.67 | **702** | 197 | **745** | 291 |
|  |  |  |  |  |  |  |  |  |
| Color PHs |  |  |  |  |  |  |  |  |
| rhed | 4 |  | 4 |  | 621 | 137 | 806 | 204 |
| ohrenge | 7 |  | 0 |  | 627 | 143 | 802 | 262 |
| yelo | 4 |  | 2 |  | 655 | 163 | 773 | 188 |
| ghrean | 6 |  | 0 |  | 675 | 237 | 754 | 205 |
| bloo | 4 |  | 4 |  | 673 | 264 | 754 | 223 |
| perpull | 7 |  | 0 |  | 663 | 215 | 758 | 232 |
| pynk | 4 |  | 2 |  | 656 | 210 | 813 | 221 |
| wyte | 4 |  | 0 |  | 600 | 133 | 778 | 191 |
| greh | 4 |  | 5 |  | 679 | 151 | 783 | 270 |
| Mean | 4.89 |  | 1.89 |  | **650** | 184 | **780** | 222 |
|  | | |  |  |  |  |  |  |
| Neutral PHs | |  |  |  |  |  |  |  |
| rapp | 4 |  | 4 |  | 647 | 166 | 758 | 183 |
| ophiss | 6 |  | 0 |  | 680 | 176 | 754 | 246 |
| yeerli | 6 |  | 0 |  | 644 | 162 | 747 | 227 |
| grayt | 5 |  | 2 |  | 754 | 232 | 761 | 268 |
| bloe | 4 |  | 7 |  | 662 | 198 | 795 | 227 |
| pupitt | 6 |  | 0 |  | 698 | 170 | 762 | 248 |
| pakk | 4 |  | 2 |  | 690 | 261 | 725 | 186 |
| wich | 4 |  | 4 |  | 654 | 145 | 759 | 204 |
| grahb | 5 |  | 0 |  | 707 | 181 | 741 | 277 |
| Mean | 4.89 |  | 2.11 |  | **682** | 188 | **772** | 229 |
|  |  |  |  |  |  |  |  |  |
| *(Appendix continues)* | | | | | | | | |
|  | | | | | | | | |
| *Appendix (continued)* | | | | | | | | |
| Word Stimulus | Length | Log Frequency HAL | Orthographic N | Phonographic N | Congruent RT | | Incongruent RT | |
|  |  |  |  |  | M | SD | M | SD |
| **Experiment 2** |  |  |  |  |  |  |  |  |
|  |  |  |  |  |  |  |  |  |
| Color Associates |  |  |  |  |  |  |  |  |
| blood | 5 | 10.86 | 4 | 1 | 603 | 150 | 695 | 214 |
| cantaloupe | 10 | 4.49 | 0 | 0 | 671 | 185 | 687 | 197 |
| yolk | 4 | 5.78 | 2 | 1 | 609 | 178 | 677 | 235 |
| bush | 4 | 9.30 | 13 | 2 | 655 | 169 | 679 | 208 |
| ocean | 5 | 9.31 | 0 | 0 | 683 | 221 | 670 | 197 |
| lilac | 5 | 5.53 | 0 | 0 | 692 | 222 | 685 | 224 |
| tongue | 6 | 9.61 | 1 | 0 | 682 | 230 | 650 | 240 |
| chalk | 5 | 7.42 | 0 | 0 | 624 | 241 | 669 | 225 |
| asphalt | 7 | 7.15 | 0 | 0 | 671 | 226 | 656 | 183 |
| Mean | 5.67 | 7.72 | 2.22 | 0.44 | **654** | 202 | **674** | 213 |
|  |  |  |  |  |  |  |  |  |
| Neutral Words |  |  |  |  |  |  |  |  |
| break | 5 | 10.77 | 6 | 0 | 607 | 159 | 655 | 205 |
| crustacean | 10 | 4.53 | 0 | 0 | 692 | 168 | 720 | 208 |
| your | 4 | 14.35 | 8 | 1 | 623 | 213 | 657 | 217 |
| bowl | 4 | 9.28 | 10 | 2 | 654 | 281 | 672 | 201 |
| odour | 5 | 5.04 | 0 | 0 | 670 | 273 | 688 | 180 |
| lapel | 5 | 5.48 | 1 | 0 | 674 | 221 | 696 | 199 |
| talked | 6 | 9.76 | 6 | 4 | 650 | 210 | 666 | 184 |
| cheque | 6 | 7.81 | 0 | 0 | 610 | 178 | 678 | 202 |
| anxious | 7 | 7.91 | 0 | 0 | 690 | 231 | 683 | 165 |
| Mean | 5.78 | 8.33 | 3.44 | 0.78 | **652** | 215 | **679** | 196 |
|  |  |  |  |  |  |  |  |  |
| Color Associate PHs | |  |  |  |  |  |  |  |
| blud | 4 |  | 4 |  | 625 | 259 | 701 | 221 |
| kantalope | 9 |  | 0 |  | 672 | 165 | 678 | 206 |
| yoak | 4 |  | 4 |  | 617 | 166 | 679 | 214 |
| boosh | 5 |  | 2 |  | 644 | 191 | 648 | 181 |
| oshin | 5 |  | 0 |  | 646 | 141 | 673 | 202 |
| lylack | 6 |  | 0 |  | 668 | 200 | 674 | 225 |
| tuhng | 5 |  | 0 |  | 653 | 153 | 658 | 189 |
| chawk | 5 |  | 2 |  | 605 | 248 | 677 | 176 |
| ashfault | 8 |  | 0 |  | 662 | 251 | 651 | 214 |
| Mean | 5.67 |  | 1.33 |  | **643** | 197 | **671** | 203 |
|  |  |  |  |  |  |  |  |  |
| Neutral Word PHs | |  |  |  |  |  |  |  |
| braik | 5 |  | 2 |  | 595 | 168 | 660 | 167 |
| krustaishin | 11 |  | 0 |  | 657 | 188 | 658 | 248 |
| yor | 3 |  | 12 |  | 617 | 199 | 674 | 174 |
| bohl | 4 |  | 8 |  | 637 | 170 | 668 | 180 |
| ohder | 5 |  | 3 |  | 660 | 246 | 681 | 197 |
| luhpel | 6 |  | 0 |  | 640 | 192 | 655 | 182 |
| tocked | 6 |  | 11 |  | 657 | 200 | 659 | 195 |
| chehk | 5 |  | 2 |  | 612 | 134 | 677 | 167 |
| angshus | 7 |  | 0 |  | 668 | 174 | 650 | 159 |
| Mean | 5.78 |  | 4.22 |  | **638** | 186 | **664** | 185 |
| *(Appendix continues)* | | | | | | | | |
|  | | | | | | | | |
| *Appendix (continued)* | | | | | | | | |
| Word Stimulus | Length | Log Frequency HAL | Orthographic N | Phonographic N | Congruent RT | | Incongruent RT | |
|  |  |  |  |  | M | SD | M | SD |
| **Experiment 3** |  |  |  |  |  |  |  |  |
| Color Words |  |  |  |  |  |  |  |  |
| red | 3 | 11.55 | 11 | 10 | 641 | 206 | 853 | 272 |
| orange | 6 | 9.51 | 1 | 0 | 661 | 182 | 831 | 262 |
| yellow | 6 | 9.87 | 3 | 3 | 686 | 182 | 820 | 288 |
| green | 5 | 11.42 | 4 | 4 | 697 | 177 | 824 | 244 |
| blue | 4 | 11.40 | 4 | 4 | 714 | 221 | 854 | 293 |
| purple | 6 | 9.15 | 0 | 0 | 701 | 203 | 803 | 289 |
| pink | 4 | 9.48 | 14 | 8 | 696 | 178 | 849 | 273 |
| white | 5 | 11.92 | 3 | 2 | 641 | 179 | 841 | 261 |
| gray | 4 | 9.45 | 8 | 4 | 696 | 171 | 838 | 248 |
| Mean | 4.78 | 10.42 | 9.00 | 3.89 | **681** | 189 | **835** | 270 |
|  | | |  |  |  |  |  |  |
| Neutral Words |  |  |  |  |  |  |  |  |
| fun | 3 | 11.234 | 11 | 10 | 708 | 187 | 776 | 213 |
| acting | 6 | 9.681 | 1 | 0 | 757 | 215 | 767 | 221 |
| dealer | 6 | 9.779 | 3 | 2 | 732 | 183 | 743 | 208 |
| story | 5 | 11.482 | 4 | 2 | 723 | 230 | 737 | 225 |
| turn | 4 | 11.404 | 5 | 4 | 725 | 216 | 752 | 231 |
| carpet | 6 | 9.136 | 0 | 0 | 787 | 228 | 734 | 176 |
| math | 4 | 9.82 | 11 | 8 | 723 | 253 | 746 | 245 |
| local | 5 | 11.848 | 3 | 1 | 733 | 194 | 754 | 208 |
| hunt | 4 | 9.639 | 8 | 4 | 761 | 212 | 741 | 201 |
| Mean | 4.78 | 10.45 | 5.11 | 3.44 | **739** | 213 | **750** | 214 |
|  |  |  |  |  |  |  |  |  |
| Color PHs |  |  |  |  |  |  |  |  |
| rhed | 4 |  | 4 |  | 620 | 157 | 846 | 216 |
| ohrenge | 7 |  | 0 |  | 653 | 171 | 856 | 215 |
| yelo | 4 |  | 2 |  | 648 | 191 | 827 | 250 |
| ghrean | 6 |  | 0 |  | 680 | 185 | 808 | 231 |
| bloo | 4 |  | 4 |  | 680 | 185 | 810 | 213 |
| perpull | 7 |  | 0 |  | 695 | 211 | 750 | 223 |
| pynk | 4 |  | 2 |  | 663 | 157 | 852 | 277 |
| wyte | 4 |  | 0 |  | 652 | 154 | 814 | 267 |
| greh | 4 |  | 5 |  | 691 | 157 | 806 | 222 |
| Mean | 4.89 |  | 1.89 |  | **665** | 174 | **818** | 235 |
|  | | |  |  |  |  |  |  |
| Neutral PHs | |  |  |  |  |  |  |  |
| phun | 4 |  | 1 |  | 673 | 142 | 745 | 196 |
| aktyng | 6 |  | 0 |  | 745 | 188 | 743 | 196 |
| deelur | 6 |  | 0 |  | 733 | 207 | 758 | 233 |
| stohree | 7 |  | 0 |  | 726 | 203 | 763 | 252 |
| ternn | 5 |  | 0 |  | 727 | 195 | 730 | 233 |
| kahrpet | 7 |  | 0 |  | 782 | 191 | 731 | 209 |
| maath | 5 |  | 0 |  | 765 | 192 | 764 | 231 |
| lowkul | 6 |  | 0 |  | 721 | 200 | 777 | 211 |
| hundt | 5 |  | 0 |  | 764 | 169 | 722 | 185 |
| Mean | 5.67 |  | 0.11 |  | **737** | 187 | **748** | 216 |
|  |  |  |  |  |  |  |  |  |
| *(Appendix continues)* | | | | | | | | |
|  | | | | | | | | |
| *Appendix (continued)* | | | | | | | | |
| Word Stimulus | Length | Log Frequency HAL | Orthographic N | Phonographic N | Congruent RT | | Incongruent RT | |
|  |  |  |  |  | M | SD | M | SD |
| **Experiment 4** |  |  |  |  |  |  |  |  |
|  |  |  |  |  |  |  |  |  |
| Color Associates |  |  |  |  |  |  |  |  |
| blood | 5 | 10.86 | 4 | 1 | 689 | 215 | 773 | 209 |
| cantaloupe | 10 | 4.49 | 0 | 0 | 748 | 195 | 782 | 208 |
| yolk | 4 | 5.78 | 2 | 1 | 741 | 167 | 774 | 265 |
| bush | 4 | 9.30 | 13 | 2 | 760 | 213 | 755 | 228 |
| ocean | 5 | 9.31 | 0 | 0 | 766 | 208 | 773 | 255 |
| lilac | 5 | 5.53 | 0 | 0 | 785 | 227 | 771 | 211 |
| tongue | 6 | 9.61 | 1 | 0 | 775 | 238 | 767 | 255 |
| chalk | 5 | 7.42 | 0 | 0 | 715 | 173 | 793 | 257 |
| asphalt | 7 | 7.15 | 0 | 0 | 774 | 215 | 773 | 206 |
| Mean | 5.67 | 7.72 | 2.22 | 0.44 | **750** | 206 | **773** | 233 |
|  |  |  |  |  |  |  |  |  |
| Neutral Words |  |  |  |  |  |  |  |  |
| peace | 5 | 10.76 | 3 | 1 | 710 | 184 | 794 | 227 |
| earthbound | 10 | 4.49 | 0 | 0 | 784 | 249 | 782 | 209 |
| hula | 4 | 5.51 | 2 | 0 | 750 | 189 | 762 | 207 |
| sing | 4 | 9.30 | 12 | 9 | 771 | 178 | 765 | 242 |
| dozen | 5 | 9.36 | 1 | 0 | 787 | 204 | 760 | 260 |
| whisk | 5 | 5.70 | 0 | 0 | 782 | 195 | 763 | 229 |
| guilty | 6 | 9.44 | 0 | 0 | 803 | 218 | 758 | 220 |
| rinse | 5 | 7.44 | 0 | 0 | 743 | 190 | 760 | 231 |
| furnace | 7 | 7.13 | 0 | 0 | 790 | 223 | 767 | 211 |
| Mean | 5.67 | 7.66 | 2 | 1.11 | **769** | 203 | **768** | 226 |
|  |  |  |  |  |  |  |  |  |
| Color Associate PHs | |  |  |  |  |  |  |  |
| blud | 4 |  | 4 |  | 732 | 221 | 773 | 209 |
| kantalope | 9 |  | 0 |  | 743 | 209 | 790 | 229 |
| yoak | 4 |  | 4 |  | 717 | 233 | 776 | 187 |
| boosh | 5 |  | 2 |  | 740 | 245 | 761 | 220 |
| oshin | 5 |  | 0 |  | 753 | 184 | 762 | 217 |
| lylack | 6 |  | 0 |  | 791 | 235 | 760 | 202 |
| tuhng | 5 |  | 0 |  | 759 | 217 | 749 | 224 |
| chawk | 5 |  | 2 |  | 729 | 216 | 780 | 234 |
| ashfault | 8 |  | 0 |  | 792 | 177 | 770 | 201 |
| Mean | 5.67 |  | 1.33 |  | **751** | 215 | **769** | 214 |
|  |  |  |  |  |  |  |  |  |
| Neutral Word PHs | |  |  |  |  |  |  |  |
| peise | 5 |  | 1 |  | 702 | 183 | 777 | 214 |
| urthbaund | 9 |  | 0 |  | 754 | 211 | 792 | 220 |
| whooluh | 7 |  | 0 |  | 742 | 232 | 761 | 208 |
| cyng | 4 |  | 0 |  | 753 | 207 | 759 | 214 |
| duhzen | 6 |  | 0 |  | 771 | 243 | 751 | 213 |
| wisck | 5 |  | 0 |  | 768 | 227 | 742 | 229 |
| giltee | 6 |  | 0 |  | 756 | 205 | 761 | 238 |
| rynce | 5 |  | 0 |  | 717 | 160 | 756 | 211 |
| phirniss | 8 |  | 0 |  | 796 | 221 | 770 | 240 |
| Mean | 6.11 |  | 0.11 |  | **751** | 210 | **763** | 221 |
|  |  |  |  |  |  |  |  |  |

*Note*. HAL = Hyperspace Analogue to Language; M = median; SD = standard deviation. Median RTs and their SDs are rounded to the nearest millisecond.
